# Watermelon rinds as cost-efficient adsorbent for acridine orange: a response surface methodological approach

**DOI:** 10.1007/s11356-021-13652-9

**Published:** 2021-04-08

**Authors:** Ahmed S. El-Shafie, Siham S. Hassan, Nuri Akther, Marwa El-Azazy

**Affiliations:** Department of Chemistry and Earth Sciences, College of Arts and Sciences, Qatar University, Doha, 2713 Qatar

**Keywords:** Acridine orange, Eco-friendly adsorbent, Wastewater, Watermelon rinds, Box–Behnken design

## Abstract

In the current investigation, watermelon rinds (WMR) have been utilized as an eco-friendly and cost-efficient adsorbent for acridine orange (AO) from contaminated water samples. Adsorption of AO onto raw (RWM) and thermally treated rinds (TTWM250 and TTWM500) has been studied. The adsorption efficiency of the three adsorbents was evaluated by measuring the % removal (%*R*) of AO and the adsorption capacity (*q*_*e*_, mg/g). Dependent variables (%*R* and *q*_*e*_) were optimized as a function of four factors: pH, sorbent dosage (AD), the concentration of AO (DC), and contact time (ST). Box–Behnken (BB) design has been utilized to obtain the optimum adsorption conditions. Prepared adsorbents have been characterized using scanning electron microscopy (SEM), Fourier-transform infrared (FT-IR), and Raman spectroscopies. The surface area of RWM, TTWM250, and TTWM500, as per the Brunauer-Emmett-Teller (BET) analysis, was 2.66, 2.93, and 5.03 m^2^/g, respectively. Equilibrium investigations suggest that Freundlich model was perfectly fit for adsorption of AO onto TTWM500. Maximum adsorption capacity (*q*_max_) of 69.44 mg/g was obtained using the Langmuir equation. Adsorption kinetics could be best described by the pseudo-second-order (PSO) model. The multi-cycle sorption-desorption study showed that TTWM500 could be regenerated with the adsorption efficiency being preserved up to 87% after six cycles.

**Supplementary Information:**

The online version contains supplementary material available at 10.1007/s11356-021-13652-9.

## Introduction

Clean-water scarcity is becoming a global concern, especially with the fast expansion of the Earth’s inhabitants and the constant climate deterioration. This expansion and the escalating anthropogenic activities such as frequent deforestation and improvised industrialization represent the main reasons behind the worldwide water crisis.

Dyes are one of the significant water contaminants. The ever-increasing use of colorants in food and beverages’ industries, cosmetics, textiles, plastics, paper, and pharmaceuticals has reached worrying levels (Mekonnen and Hoekstra 2016; Liu et al. 2017; Gleick and Iceland 2018). Even traces of dyes (as low as 1 ppm) can highly affect a substantial volume of water, an issue that consequently increases the magnitude of the problem by affecting the aquatic creatures and human health. Statistics show that approximately 10^6^ tons of dyes are consumed worldwide per year and the quantity of dyes discharged into water sources represents 2% of this amount (Forgacs et al. 2004; Kant 2012; Ghaly et al. 2014; Basu et al. 2014; Jawad et al. 2019; El-Azazy et al. 2020; Hassan et al. 2020; El-Azazy et al. 2019a, 2019b; Prasad and Santhi 2012).

**Scheme 1:**
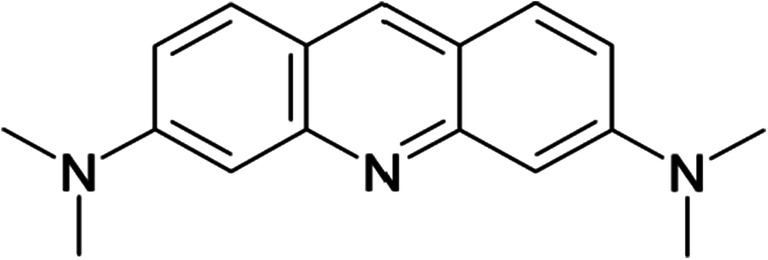
Structure of acridine orange (AO)

Acridine orange (AO) (IUPAC name: 3-N,3-N,6-N,6-N-tetramethylacridine-3,6-diamine, C_17_H_20_CIN_3_) is a xenobiotic, nucleic acid-selective, and cell-permeable fluorescent cationic dye. AO is broadly used for biological staining applications, lithography, block-printing, and coloring leather products (Scheme 1). According to the “safety data sheet” of AO, this dye’s remarkable hazards include lethality, photo-dynamicity, and mutagenicity (Iessi et al. 2017; IARC 1978).

In general, wastewater treatment technologies can be categorized into chemical, physical, biological, and combinatorial (two or more of these approaches). Each of these techniques has its pros and cons. For example, chemical and physical approaches and unless being coupled to response surface methodology (RSM) would be time and resources consuming. Moreover, these schemes generally suffer from drawbacks such as low % removal, high cost, sludge-formation, and complicated setup (Camargo et al. 2016; Verma et al. 2012; Montes-Atenas and Valenzuela 2017; Hai et al. 2007; Geaniyu et al. 2015; El-Gendy et al. 2020; Al-Saad et al. 2019; El-Azazy et al. 2019c, d). Adsorption is a promising technique that offers many advantages such as being convenient with simple setup, low cost, high removal efficiency, and good output quality.

Low-cost adsorbents represent an emerging class of adsorbents. As their name implies, these adsorbents are cost-effective, eco-friendly, and readily available. Moreover, using these wastes has a dual environmental benefit, for both waste management and wastewater treatment. Several investigations on the valorization of agro-wastes for the removal of water contaminants can be found in the literature (Basu et al. 2014; Jawad et al. 2019; El-Azazy et al. 2020; Hassan et al. 2020; El-Azazy et al. 2019a, 2019b, 2019c, 2019d; Prasad and Santhi 2012; El-Gendy et al. 2020; Al-Saad et al. 2019).

Watermelon (*Citrullus lanatus*) is a well-known summer-fruit in which the rind covers approximately one-third of the total fruit mass. China alone produces around 2.3 × 10^7^ tons of rinds per year (Chen et al. 2017). The rind biomass is considered waste and has no commercial value. As a lignocellulosic material, watermelon rinds (WMR) are rich in macromolecular polymers (e.g., cellulose, pectin, carotenoids), which in turn possess a variety of functional moieties and would facilitate the adsorption of pollutants from wastewater (Chen et al. 2017; Lakshmipathy and Sarada 2013; Lakshmipathy and Sarada 2015; Alexander et al. 2015; Husein et al. 2017; Masoudian et al. 2019; Gupta and Gogate 2016; Memon et al. 2008; Li et al. 2019; Benkaddour et al. 2018). Several studies have explored the adsorption efficiency of WM (specially rinds and seeds both raw and activated) for various contaminants. Samples of these applications are summarized in Table S1 (Masoudian et al. 2019; Gupta and Gogate 2016; Memon et al. 2008; Li et al. 2019; Benkaddour et al. 2018).

Literature survey shows that the adsorption efficiency of is affected by different factors such as pH, adsorbent dosage (AD), dye concentration (DC), and the contact time between the adsorbent and the contaminant (ST). Yet, the majority of the reported approaches (Table S1) are one-factor-at-a-time (OFAT) based, where one variable is altered while the remainder is kept constant. This technique, and in addition to the deterioration of the method greenness, does not draw a full picture of variable-variable interactions (Elazazy 2017). Therefore, it was imperative to develop an approach that surmounts these detriments and produces data that can be handled with a great deal of confidence. Box–Behnken (BB) design was therefore selected for the current endeavor. Two responses, % removal of AO (%*R*) and adsorption capacity of WMR (*q*_*e*_, mg/g), will be determined and maximized as a function of the four previously mentioned variables (Elazazy 2017; Elmoubarki et al. 2017; Elazazy et al. 2018; El-Azazy et al. 2021; Oladipo et al. 2015).

Prepared adsorbents will be characterized using scanning electron microscopy (SEM), sorption-desorption of N_2_ on the surface, Fourier transform-infrared (FT-IR), and Raman spectroscopies. Thermal stability will be assessed using thermogravimetric analysis (TGA). Identifying the adsorption mechanism will be accomplished by analyzing the adsorption equilibrium and kinetics.

## Materials and methods

### Materials and reagents

Watermelon rinds (WMR) were collected as a waste material (to be discarded) from the shopping centers, Doha-Qatar. Collected rinds were washed with distilled water to eliminate any dirt. All chemicals used were of the analytical grade. Sodium hydroxide, sulfuric acid, nitric acid, sodium carbonate, hydrochloric acid, and sodium tetraborate-10-hydrate were the products of Sigma-Aldrich (Darmstadt, Germany). Adsorbate tested was acridine orange dye (AO, Fluka Chemicals, Germany). Ultrapure water (18.2 MΩ) was used to prepare and dilute dye solutions. A stock solution of 100 mg/L of AO was made in deionized water. The pH of dye solutions was attuned using a mixture of sodium tetraborate-10-hydrate (Na_2_B_4_O_7_.10H_2_O, 50 mM) and 0.1 M NaOH or 0.1 M HCl. All experimental runs were carried out at room temperature.

### Instrumentation and software

A Thermo Scientific centrifuge (SL8 Benchtop, Thermo Scientific, MA, USA) was operated to separate the supernatant. The amount of adsorbed AO was determined using a UV/Vis spectrophotometer (Agilent diode-array, CA, USA) equipped with identical 10-mm quartz cells. For pH measurements, a pH meter (Jenway, Staffordshire, UK) was used. Study of surface morphology and composition was performed using SEM (FEI, Quanta 200, Thermo Scientific, MA, USA). Existence of surface functionalities was performed using FT-IR analysis (Bruker Alpha, MA, USA). Conversion of WMR into carbonaceous biomass was investigated using Raman spectroscopic analysis. Spectrum was acquired in the range of 50–3500 cm^−1^ operating a DXRTM 2 Raman microscope (Thermo Scientific, MA, USA), with a laser beam at 532 nm as excitation source and 10 mW power. The prepared adsorbents’ thermal stability was assessed in the range of 50–800 °C using a thermal gravimetric analyzer (TGA, PerkinElmer-TGA400). To determine the surface area and pore size, samples were degassed and N_2_ adsorption-desorption was monitored utilizing a Micromeritics ASAP2020 Accelerated Surface Area and Porosimetry System. The N_2_ adsorption-desorption isotherms obtained at 77 K and using the Brunauer Emmett-Teller (BET) equation were utilized to determine surface area. Pore volume was ascertained employing the t-plots and Barrett–Joyner–Halenda (BJH) equation.

Minitab®19 software was used for composing and analyzing the Box–Behnken (BB) design. The software was procured from Minitab (Minitab Inc., State College, PA, USA).

### Preparation of AO solutions

Contaminated water samples (artificially, 100 mg/L stock solutions) were made by the dissolution of the appropriate amounts of AO in deionized water. Serialized dilutions of AO were made in the same solvent, and the pH was tuned to the required value. Calibration curves of AO at the three-pH levels ventured in the design structure (low, central, and high, Table 1) were assembled using different concentrations of AO at 419 nm.

**Table 1:** Process factors and their boundaries

Factors	High (+)	Mid (0)	Low (−)
pH value (pH, A, pH unit)	11.0	7.5	4.0
Adsorbent dosage (AD, B, mg/15 mL)	125	75	25
Dye concentration (DC, C, mg/L)	25	15	5
Contact time (ST, D, min)	240	130	20

### Preparation of the adsorbents

For this step, WMR was chopped into smaller pieces (nearly 1 cm × 1 cm) and further washed with deionized water to eliminate dust and impurities. Cut pieces were dried out in the oven at 80 °C for six consecutive days. Dry rinds were broken up into smaller pieces, powdered, and sieved using a 1-mm mesh size sieve. Crushed rinds were apportioned into two parts: one-half for testing the raw sample’s efficiency (raw, RWM) and the other half for testing as thermally treated samples at two different temperatures, 250 °C and 500 °C. The crushed rinds were then placed in a covered crucible and were burnt in a furnace at the mentioned temperatures for 2 h. Soon after, the crucibles were left to cool down before collecting the burnt samples, and the obtained portions were labeled as TTWM250 and TTWM500.

### Assessment of adsorption competency of the prepared WMR

The adsorption capability of the three adsorbents was evaluated by contrasting the values of the %*R* and *q*_*e*_ using Eqs. 1 and 2, respectively, and operating the conditions mentioned in Table 1. TTWM500 showed the best performance in terms of both responses (Table 1).

1 $$ \%R=\frac{\left({C}_o-\kern0.5em {C}_e\right)\ }{C_o}\kern0.5em \times 100\% $$

2 $$ {q}_e=\frac{\left({C}_o-\kern0.5em {C}_e\right)\ }{W}\ V $$

In the above equations, *q*_*e*_ stands for adsorption capacity, *C*_0_ and *C*_*e*_ stand for the primary and equilibrium concentrations of AO in solution (mg/L), *V* is the volume of the solution (L), *W* is the mass of the adsorbent in grams. To determine the effect of experimental parameters on the adsorption process, the experimental structure shown in Table 2 was followed, and the two responses were determined using Eqs. 1 and 2. The absorbance of the supernatant for each experiment was measured against a blank prepared simultaneously omitting the AO.

**Table 2:** Performance of tested adsorbents. Assessment was carried out using a variable blend of pH = 7.00 ± 0.20, DC = 20 mg/L, AD = 100 mg/15 mL, and ST = 30 min

Adsorbent type	%R^*^	*q*_*e*_ (mg/g)^**^
RWM	77.17	2.31
TTWM250	96.21	2.88
TTWM500	98.32	2.94

^*,**^Calculated using Eqs. 1 and 2, respectively

### Response surface methodology (RSM)

TTWM500, and following the comparison shown in Table 2, was the adsorbent of choice for the subsequent investigations. A response surface methodological approach, BB design was used to investigate the impact of the different variables on the measured responses. Two responses, %*R* and *q*_*e*_, were optimized as a function of four factors. The target was to maximize both responses. In this itinerary, 28 experimental runs (including four central points) were conducted over three blocks. Regression equations were obtained by including blocks in the model. The design structure is shown in Table 3.

**Table 3:** Experimental structure of the BB design as generated by Minitab® 19

Run#	Block	pH	AD	DC	ST	%*R* (obs.*)	%*R* (pred.^**^)	Er^***^	*q*_*e*_ (obs.*)	*q*_*e*_ (pred.^**^)	Er^***^
01	2	11(+)	75(0)	15(0)	240(+)	88.23	89.05	0.82	4.41	4.43	0.02
02	2	7.5(0)	25(−)	25(+)	130(0)	88.21	86.70	1.51	21.48	20.60	0.88
03	2	4(−)	75(0)	15(0)	20(−)	94.50	93.33	1.17	4.71	4.52	0.19
04	2	4(−)	75(0)	15(0)	240(+)	98.54	98.06	0.48	4.93	4.71	0.22
05	2	7.5(0)	25(−)	5(−)	130(0)	93.74	90.77	2.97	4.66	4.35	0.31
06	2	7.5(0)	75(0)	15(0)	130(0)	94.93	95.06	0.13	4.66	4.66	0.00
07	2	11(+)	75(0)	15(0)	20(−)	86.56	86.65	0.09	4.23	4.27	0.04
08	2	7.5(0)	125(+)	5(−)	130(0)	93.78	94.33	0.55	0.94	0.94	0.00
09	2	7.5(0)	125(+)	25(+)	130(0)	97.71	98.13	0.42	4.88	5.03	0.15
10	1	7.5(0)	75(0)	5(−)	20(−)	93.74	93.36	0.38	1.56	1.52	0.04
11	1	7.5(0)	75(0)	15(0)	130(0)	95.84	95.06	0.78	4.73	4.66	0.07
12	1	11(+)	125(+)	15(0)	130(0)	87.72	81.54	6.18	2.63	2.50	0.13
13	1	7.5(0)	75(0)	25(+)	240(+)	97.08	97.09	0.01	8.04	7.97	0.07
14	1	4(−)	125(+)	15(0)	130(0)	98.46	99.40	0.94	2.94	3.09	0.15
15	1	7.5(0)	75(0)	15(0)	130(0)	94.54	95.06	0.52	4.61	4.66	0.05
16	1	7.5(0)	75(0)	25(+)	20(−)	95.63	95.95	0.32	7.86	7.76	0.10
17	1	7.5(0)	75(0)	5(−)	240(+)	99.13	98.56	0.57	1.63	1.60	0.03
18	1	4(−)	25(−)	15(0)	130(0)	75.33	81.88	6.55	11.30	11.53	0.23
19	1	11(+)	25(−)	15(0)	130(0)	91.89	83.74	8.15	13.78	12.69	1.09
20	3	4(−)	75(0)	25(+)	130(0)	90.86	90.45	0.41	7.39	7.32	0.07
21	3	7.5(0)	25(−)	15(0)	20(−)	76.14	88.34	12.2	11.20	12.21	1.01
22	3	7.5(0)	75(0)	15(0)	130(0)	94.78	95.06	0.28	4.70	4.66	0.04
23	3	7.5(0)	125(+)	15(0)	240(+)	99.96	98.95	1.01	2.97	2.92	0.05
24	3	4(−)	75(0)	5(−)	130(0)	97.76	98.01	0.25	1.61	1.65	0.04
25	3	7.5(0)	125(+)	15(0)	20(−)	98.71	95.95	2.76	2.93	2.87	0.06
26	3	11(+)	75(0)	25(+)	130(0)	90.88	91.45	0.57	7.51	7.83	0.32
27	3	7.5(0)	25(−)	15(0)	240(+)	81.66	92.39	10.73	11.85	12.93	1.08
28	3	11(+)	75(0)	5(−)	130(0)	76.30	79.99	3.69	1.27	1.37	0.10

^*^Obs., experimental values; ^**^Pred., predicted values following the process of response transformation. ^***^Er, absolute error = |(Obs − Pred.)|

### Equilibrium and kinetics investigation

To get the equilibrium features of the adsorption of AO onto WMR, a 1000 mg/L solution of AO was formulated. Additional dilutions of the stock solution (5–400 mg/L) were prepared using the same solvent and the pH was attuned to a value of 7.00 ± 0.20 using the mentioned borate-HCl or borate-NaOH solutions. Quantities of TTWM500 (0.100 ± 0.005 g) were inserted into 13 mL of the formerly made solutions, and then the solution was stirred at a speed of 150 rpm for 2 h and then filtered. Investigation of the adsorption kinetics was carried out using 200 mL of AO solution (100 mg/L, pH 4.00 ± 0.20) and ~ 1 g of TTWM500 with shaking. One sample was taken at a time range of around 1 min and over a total time span of 30 min. Investigations of equilibrium and kinetics were conducted at room temperature.

### Desorption and reusability studies

To explore the potential of adsorbent reusability, TTWM500 (2.5 g) was first equilibrated with 1 L of 15 mg/L dye solution over a period of 4 h at room temperature. The mixture was then filtered. The adsorbent was washed with distilled water to remove any non-adsorbed traces of the AO, and then dried in the oven at 80 °C overnight. The eluents used in the current study were 0.1 M of HCl, H_2_SO_4_, HNO_3_, Na_2_CO_3_, NaOH, and H_2_O. The desorption experiment was performed by mixing 100 mg of the AO-loaded adsorbent with 10 mL of the eluent and stirring at 150 rpm for 1 h. The mixture was filtered, and the absorbance of the filtrate was measured using the UV-Vis spectrophotometer. Each of the desorption experiments was repeated three times and the average values of desorbed amount were plotted. Error bars were used to express the standard deviation between the replicate measurements.

Recovery studies were carried using 0.1 M NaOH solution. Prior to that, an amount of 200 mg of TTWM500 was equilibrated with 40 mL of 15 mg/L AO solution (pH 4.0 ± 0.2) for 4 h at room temperature. The obtained mixture was then filtered, and the absorbance of the filtrate was measured at 491 nm. The loaded adsorbent was washed with distilled water and then left in contact with 20 mL of 0.1 M NaOH for 1 h. The adsorbent was washed and dried in the next step at 80 °C for 2 h and this adsorbent was used again for a second adsorption cycle. This process was renewed six times, and in each cycle, the removal efficiency (%*R*) was determined.

## Results and discussion

### Box–Behnken (BB) design

The main endeavor we are taking herein is to maximize the adsorption efficiency and the removal capability of WMR. Concomitantly, conservation of method greenness via recycling of the WMR as an agro-waste into a waste removing *model* adsorbent using a green approach was the task undertaken. These targets were tackled using BB design as an approach. As a response surface design, BB design can proficiently determine the first- and second-order constants and consequently provide an idea about the experimental response surface’s shape. BB design is a straightforward design with no involvement of a precursor factorial design. As a multivariate approach, this design is a cost-effective option as it comprises fewer design points and hereafter a small number of experimental runs contrasted to other RSM designs (Elazazy 2017). The experimental structure is displayed in Table 3.

#### Data analysis and development of regression models

**Fig. 1:**
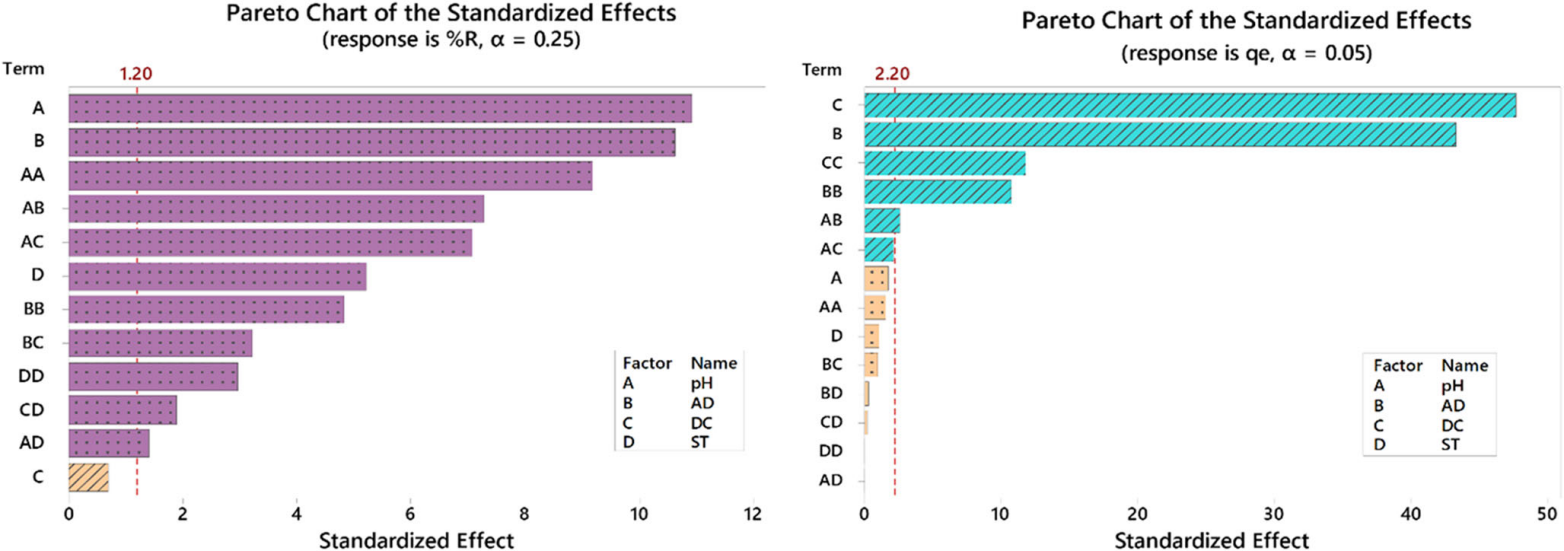
Pareto chart of standardized effects for both responses (%*R* — left panel and *q*_*e*_ — right panel)

Quality charts were utilized to confirm variable significance. Pareto chart of the standardized effects was employed as a tool to determine the statistically significant factors. Figure 1—left panel—shows that while %*R* was most affected by pH (A) followed by the AD (B), the DC (C) was not statistically substantial. However, the right panel shows that DC (C) in the case of *q*_*e*_ was the most statistically weighty factor.

**Fig. 2:**
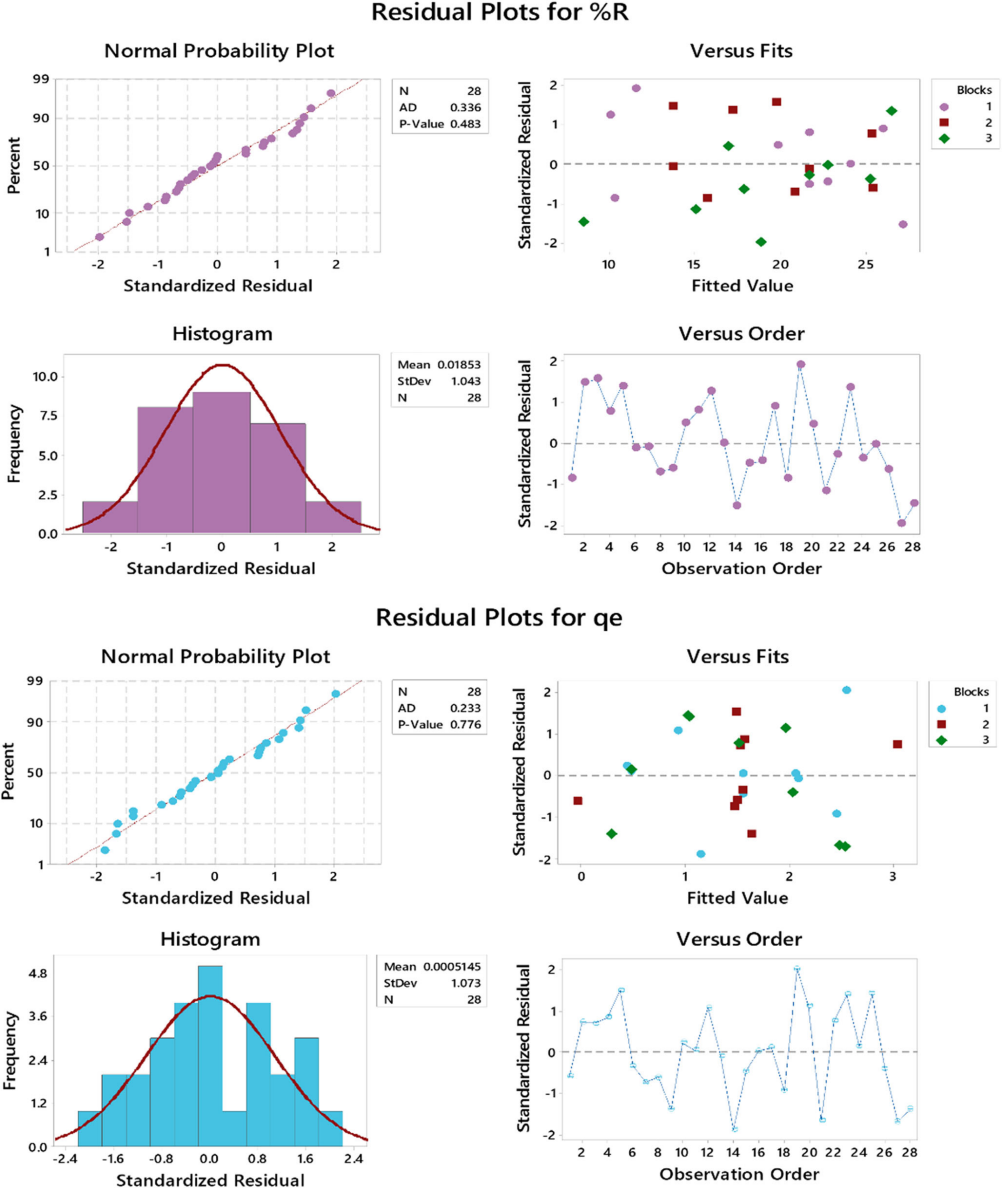
Residual plots of both responses (%*R* — upper panel and *q*_*e*_ — lower panel)

Figure 2 demonstrates the residual plots (four types of charts in each panel: normal probability plots, versus fits, histogram of residuals, and versus order). Residual plots were used to confirm that the original assumption of ordinary least square was fulfilled, and so is the goodness-of-fit in regression. Plots shown for both responses confirm that data are coming from a normal population where residuals look normally distributed at constant variance with almost no outliers and no correlation between residuals.

The outcome of using BB design is the regression models shown below (Eqs. 3 and 4). These two models were obtained following Box–Cox transformation with a forward selection of terms in the case of %*R* (Box and Cox 1964; Antony 2003; Bruns et al. 2006). Moreover, these equations could be used to get the magnitude and direction of tested variables by comparing their sign and the coefficient, respectively. The overall effect of a variable could be gauged considering the contributions from linear, quadratic, and two-way interactions.

3 $$ \left(\%R\hat{\mkern6mu} \lambda -1\right)/\left(\lambda \times g\hat{\mkern6mu} \left(\lambda -1\right)\right)=-2.69+4.789\ \mathrm{pH}+0.3548\ \mathrm{AD}-1.085\ \mathrm{DC}+0.0191\ \mathrm{ST}-0.3708\ {\mathrm{pH}}^2-0.000955\ {\mathrm{AD}}^2+0.000121\ {\mathrm{ST}}^2-0.02604\ \mathrm{pH}\times \mathrm{AD}+0.1265\ \mathrm{pH}\times \mathrm{DC}-0.00230\ \mathrm{pH}\times \mathrm{ST}+0.00403\ \mathrm{AD}\times \mathrm{DC}-0.001082\ \mathrm{DC}\times \mathrm{ST},\left(\lambda =5,g=92.0315\ \mathrm{is}\ \mathrm{the}\ \mathrm{geometric}\ \mathrm{mean}\ \mathrm{of}\%R\right),\left[{R}^2=97.10\%,{R}^2\left(\mathrm{adj}\right)=94.78\%,{R}^2\left(\mathrm{pred}\right)=88.40\%\right] $$

4 $$ \mathit{\ln}\left({\mathrm{q}}_{\mathrm{e}}\right)= 1.223+ 0.0454\  pH- 0.02750\  AD+ 0.1487\  DC+ 0.000423\  ST- 0.00317\ {pH}^2+ 0.000103\ {AD}^2-0.002844\ {\mathrm{DC}}^2-0.000000\ {\mathrm{ST}}^2-0.000443\ \mathrm{pH}\times \mathrm{AD}+0.001818\ \mathrm{pH}\times \mathrm{DC}-0.000002\ \mathrm{pH}\times \mathrm{ST}+0.000060\ \mathrm{AD}\times \mathrm{DC}-0.000002\ \mathrm{AD}\times \mathrm{ST}-0.000005\ \mathrm{DC}\times \mathrm{ST},\left[{\mathrm{R}}^2=99.76\%,{\mathrm{R}}^2\left(\mathrm{adj}\right)=99.40\%,{\mathrm{R}}^2\left(\mathrm{pred}\right)=98.03\%\right] $$

As shown in Table 3, the measured responses (observed) as well as the responses anticipated by the regression model (predicted) are compared and the comparison is given in terms of the absolute error (Er). Results show good agreement between both (Table 3). Moreover, the regression model prediction capability was confirmed by the high values of *R*^2^(pred).

#### Analysis of variance (ANOVA)

Parallel to the use of quality charts, analysis of variance (ANOVA) was employed to corroborate the variable significance at 95% CI. Results of the ANOVA for both responses are summarized in Table 4. Variable significance is verified when *P*-values are ˂ 0.05. For example, linear and squared variables: pH, AD, and ST as well as the two-way interaction effects of pH⨯DC, pH⨯AD, and DC⨯AD were statistically significant for the %*R* with *P*-values being ˂ 0.05. Lack-of-fit values for both responses were statistically insignificant implying the goodness-of-fit of the proposed models (Elazazy 2017; Box and Cox 1964; Antony 2003; Bruns et al. 2006).

**Table 4:** Results of the ANOVA for the assessed responses

Response		*q*_*e*_				%*R*				
Source	DF*	Adj SS*	Adj MS*	*F*-value*	*P*-value*	DF*	Adj SS*	Adj MS*	*F*-value*	*P*-value*
Model	16	15.5658	0.97286	281.46	0.000	12	787.334	65.611	41.88	0.000
Blocks	2	0.0114	0.00568	1.64	0.238					
Linear	4	14.3229	3.58073	1035.96	0.000	4	407.172	101.793	64.98	0.000
pH	1	0.0105	0.01047	3.03	0.110	1	186.911	186.911	119.32	0.000
AD	1	6.4724	6.47244	1872.58	0.000	1	176.911	176.911	112.94	0.000
DC	1	7.8358	7.83578	2267.02	0.000	1	0.784	0.784	0.50	0.490
ST	1	0.0042	0.00423	1.22	0.292	1	42.566	42.566	27.17	0.000
Square	4	1.1863	0.29658	85.80	0.000	3	193.680	64.560	41.21	0.000
pH^2^	1	0.0090	0.00900	2.60	0.135	1	132.060	132.060	84.30	0.000
AD^2^	1	0.3966	0.39655	114.73	0.000	1	36.458	36.458	23.27	0.000
DC^2^	1	0.4821	0.48214	139.49	0.000					
ST^2^	1	0.0000	0.00001	0.00	0.970	1	13.729	13.729	8.76	0.010
2-way interactions	6	0.0444	0.00740	2.14	0.130	5	186.482	37.296	23.81	0.000
pH⨯AD	1	0.0241	0.02406	6.96	0.023	1	83.053	83.053	53.02	0.000
pH⨯DC	1	0.0162	0.01620	4.69	0.053	1	78.385	78.385	50.04	0.000
pH⨯ST	1	0.0000	0.00000	0.00	0.979	1	3.138	3.138	2.00	0.177
AD⨯DC	1	0.0036	0.00361	1.04	0.329	1	16.243	16.243	10.37	0.006
AD⨯ST	1	0.0004	0.00041	0.12	0.737					
DC⨯ST	1	0.0001	0.00013	0.04	0.847	1	5.662	5.662	3.61	0.077
Error	11	0.0380	0.00346			15	23.497	1.566		
Lack-of-fit	10	0.0377	0.00377	12.53	0.217	14	22.394	1.600	1.45	0.580
Pure error	1	0.0003	0.00030			1	1.103	1.103		
Total	27	15.6038				27	810.831			

*DF, degrees of freedom; *Adj SS*, adjusted sums of squares; and *Adj MS*, adjusted mean of squares

#### Contour (2D), surface (3D), and optimization plots

**Fig. 3:**
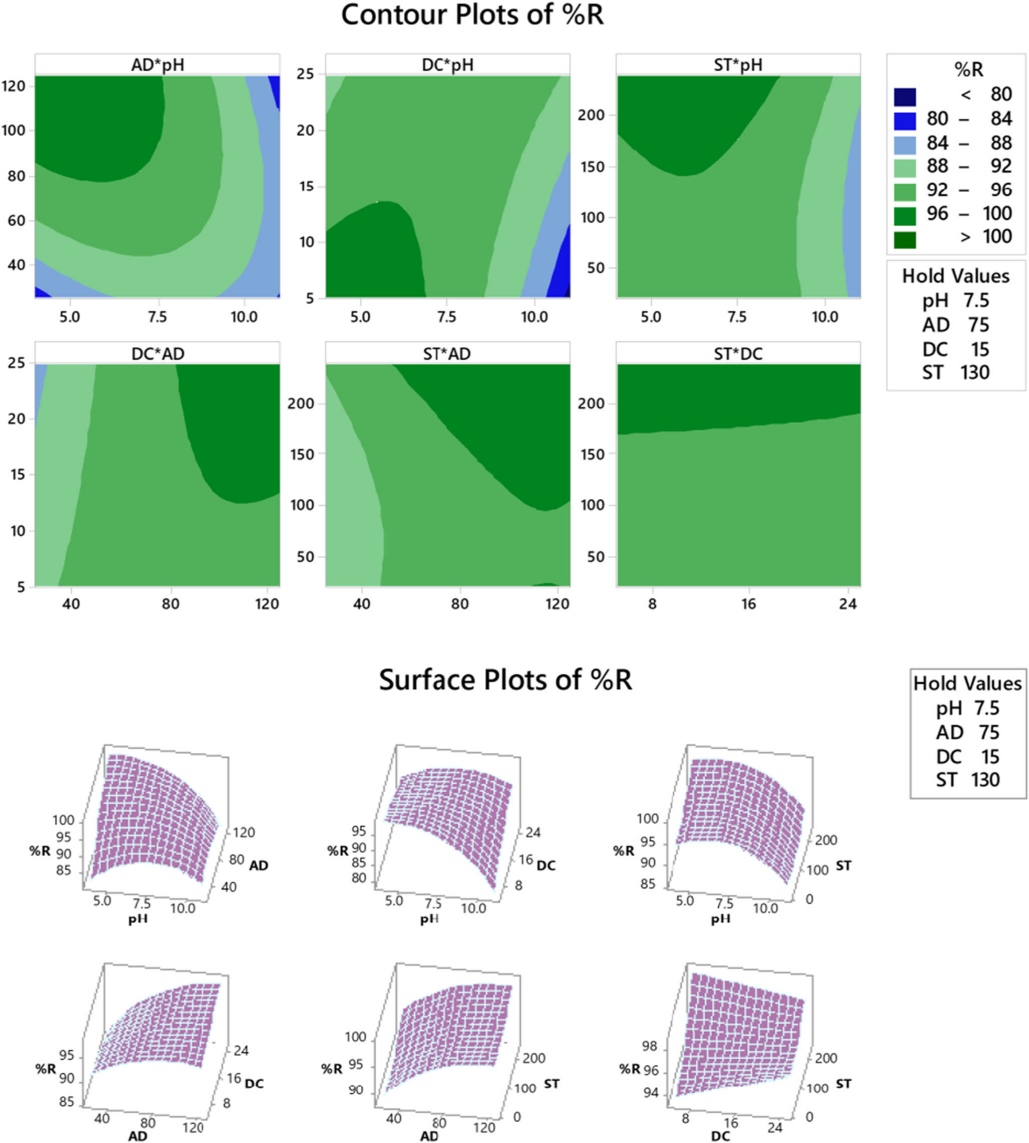
Contour (upper panel) and surface (lower panel) plots of %*R*

Contour plots disclose a three-dimensional surface on a two-dimensional plane, where the two predictors *X* and *Y* are shown on the y-axis while a response variable *Z* is displayed as contours. In Fig. 3 — upper panel — the densest green region denotes an area with the ultimate response. The 2D plot of %*R* for example displays 100% removal of AO at a pH range of 5–7.5 and an AD of 80–120 mg/50 mL. On the other hand, surface plots are graphs of three-dimensional data that illustrate a functional relationship between a designated dependent variable (*Z*), and two independent variables (*X* and *Y*), rather than showing the individual data points. Figure 3 — lower panel — exhibits the 3D arrangement. Same conclusions as in the case of the contour plots can be derived in the case of pH–AD 3D-plot, where the maximum curvature denotes the maximum %*R*.

Optimization plots — figures are not shown — display the best level for each factor as well as the best factorial blend to maximize each response. The favorability of the blend is denoted by the value of the individual desirability function (*d*). Overall, as the value of *d* gets close to 1.000, the factorial blend is then more favorable (Elazazy 2017; Box and Cox 1964; Antony 2003; Bruns et al. 2006). Optimum conditions that maximize the %*R* were pH = 4.0, AD = 125 mg/50 mL, DC = 5 mg/L, and ST = 240 min. This blend could achieve a removal of 100% and a *d* = 1.000. In the case of *q*_*e*_, however, a blend of pH = 11, AD = 25 mg/50 mL, DC = 25 mg/L, and ST = 240 min could achieve an adsorption capacity of 22.64 mg/g with a *d* = 1.000.

### Adsorbent characterization

#### SEM and BET analyses

**Fig. 4:**
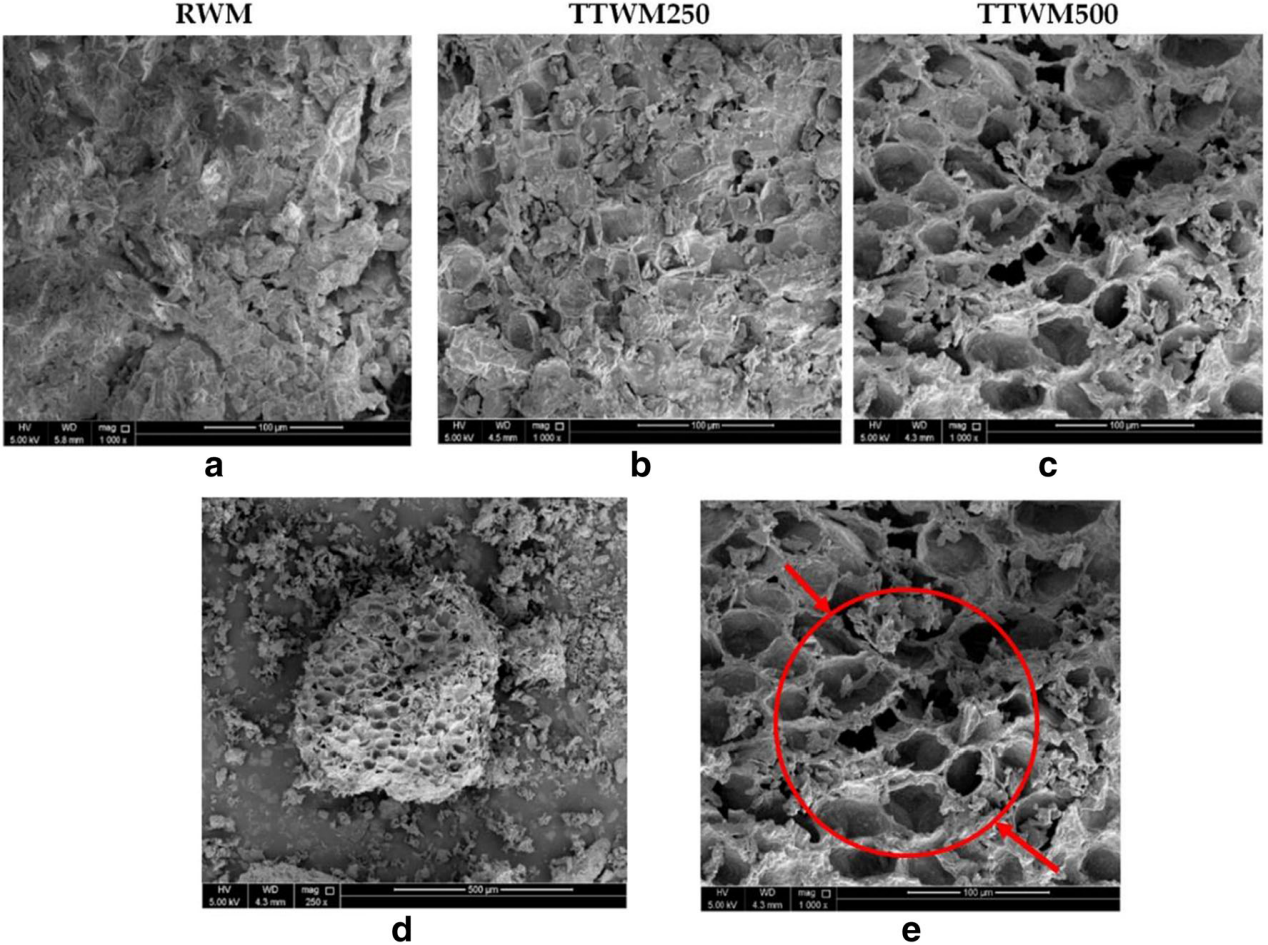
SEM images of the three tested adsorbents (**a** RWM, **b** TTWM250, **c** TTWM500) at × 1000 magnification, and **d** TTWM 500 (× 250) and **e** TTWM 500 (× 500)

The structural features of the surface of the three adsorbents were studied using SEM and BET analyses. Figures 4a, 4b, and 4c show the SEM images of RWM, TTWM250, and TTWM500 at × 1000 magnification, respectively. The SEM micrograph of RWM (Fig. 4a) shows no formation of pores with a homogenous, smooth surface, in contrast to TTWM250 and TTWM500; Figs. 4b and 4c show clear formation of pores. This could be ascribed to the thermal activation process and the conversion of the biomass into a carbonaceous material with an advanced pore structure. Other images for TTWM500 at magnification × 250 and × 1000 are shown in Fig. 4 — lower panel. Moreover, the SEM images show that TTWM500 has a more advanced intra-pore structure compared to TTWM250 (Li et al. 2019).

**Fig. 5:**
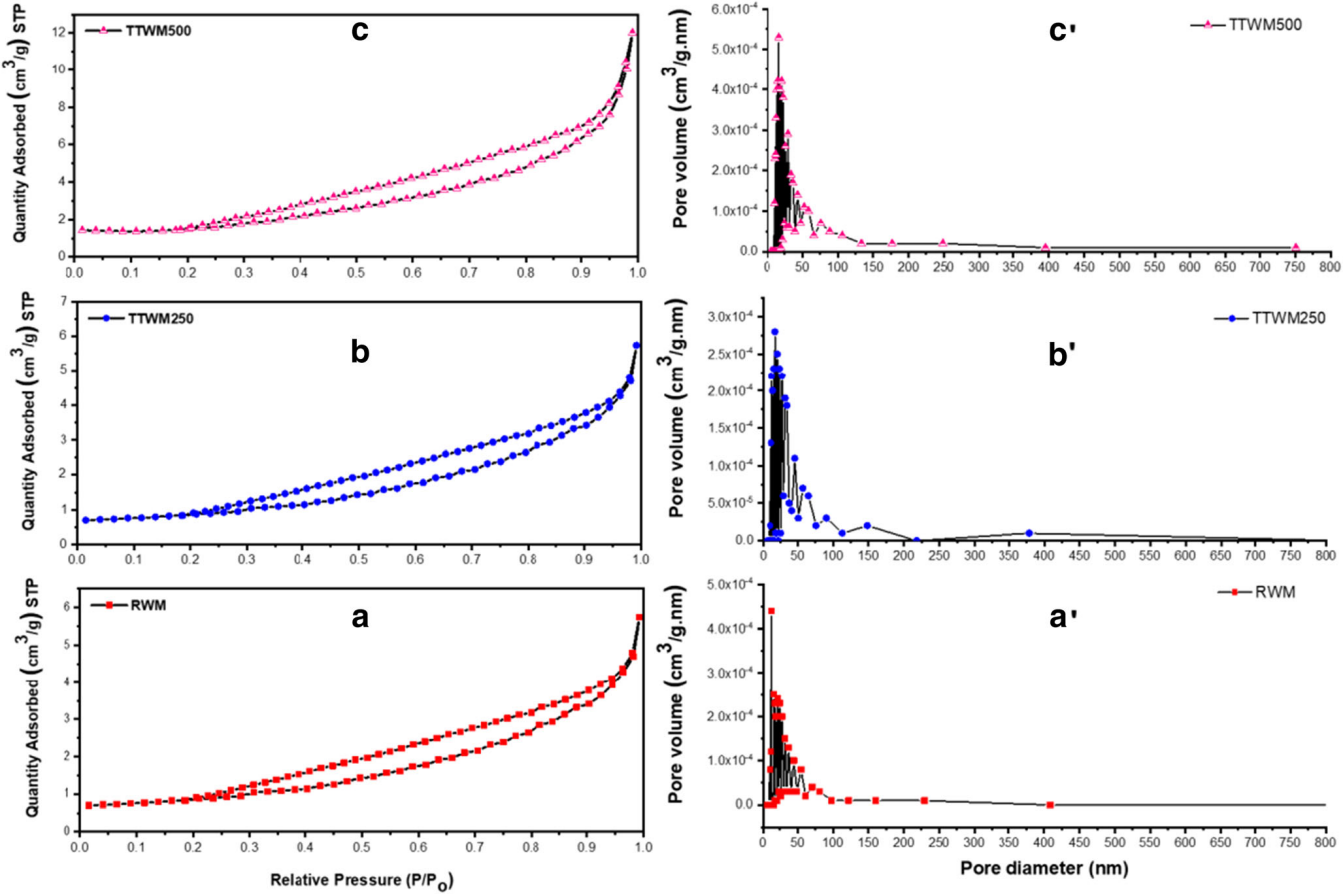
N_2_ adsorption-desorption isotherms for the tested adsorbents

BET analysis results of the three adsorbents are reported in Table 5. Obtained results show that the surface areas of the raw and biochar adsorbents are not that high compared to the previously reported surface areas of other waste-derived carbonaceous materials (Al-Saad et al. 2019; El-Azazy et al. 2019c, 2019d). However, in comparison to the untreated WM-derived adsorbents (Table S1), the obtained surface area is reasonable (Li et al. 2019). TTWM500 showed the highest surface area, pore-volume, and pore radius among the tested adsorbents. The N_2_ adsorption-desorption isotherms for the three adsorbents are shown in Fig. 5. As per the IUPAC classifications for the porous materials at 1 atm and 77 K, the RWM surface is mainly microporous with few mesopores. At the same time, TTWM250 and TTWM500 are mostly mesoporous with fewer macropores (Rouqueroltd et al. 1994). The three adsorbents show type III adsorption isotherms with a significant hysteresis H3 — type loop, indicating that multilayer adsorption is taking place on plate-like pores.

**Table 5:** BET analysis of the three adsorbents

Adsorbent	BET surface area (m^2^/g)	Langmuir surface area (m^2^/g)	Total pore volume (cm^3^/g)	Average pore radius (Å)
RWM	2.66	3.58	0.009	67.2
TTWM250	2.93	4.11	0.010	70.4
TTWM500	5.03	5.74	0.019	74.2

#### FT-IR and Raman spectroscopic analyses

**Fig. 6:**
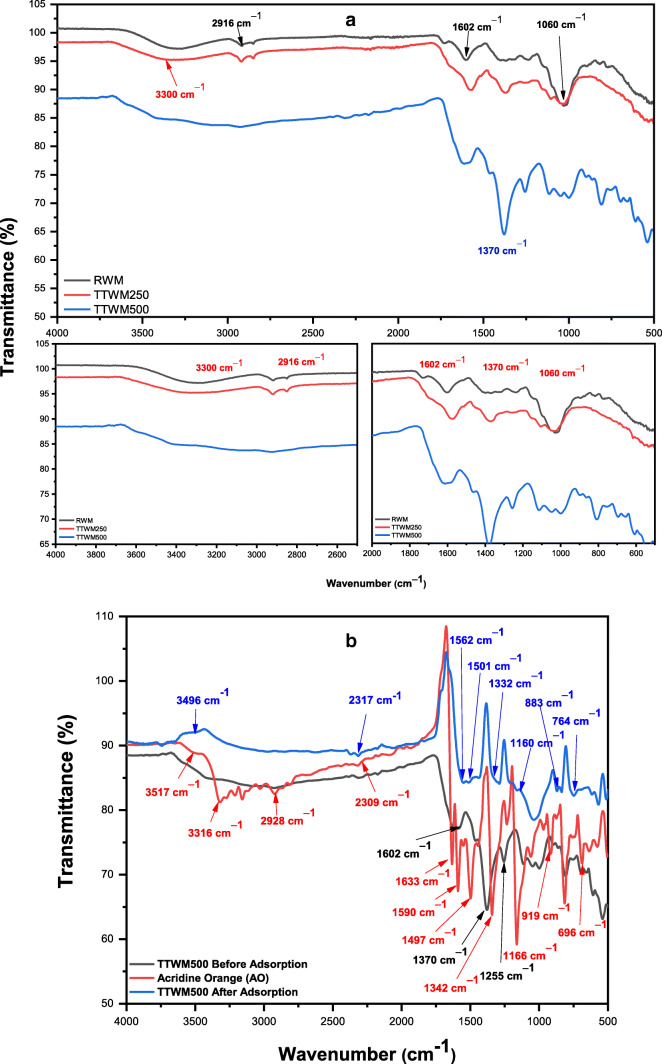
**a** FT-IR spectra of the three adsorbents. **b** FT-IR spectra of the TTWM500 before and after adsorption of acridine orange (AO)

Figure 6a shows the FT-IR spectra of the as-prepared adsorbents, while Fig. 6b shows a comparison between the spectra of TTWM500 before and after adsorption of AO as well as the spectrum of the free AO. As shown in Fig. 6a — upper panel (RWM) — a broad peak can be observed at 3300 cm^−1^ and could be assigned to the –OH group that might be coming from physical and crystalline water. This peak is also observed in TTWM250 with lower intensity probably due to thermal treatment, and it has almost completely disappeared in TTWM500. A small peak can be observed at 2916 cm^−1^ in RWM, corresponding to the C–H symmetric and asymmetric stretching vibrations. The same peak could be observed in TTWM250 with lower intensity and it completely disappeared in TTWM500. The peak (in all three adsorbents) at around 1600 cm^−1^ could be assigned to C=C stretching. Moreover, the spectra show two peaks at 1379 cm^−1^, which corresponds to methyl group vibration, and 1060 cm^−1^, which could be ascribed to C=O vibration of carboxylic acid, aldehyde, and ketone (Abbas and Ahmed 2016; Reddy et al. 2014; López-velandia et al. 2014; Jawad et al. 2018; Chaudhari and Singhal 2015). The obtained data confirm the existence of different functional moieties on the surface of RWM with a lower intensity in the case of TTWM300 and the same groups almost disappear in TTWM500 due to the thermal treatment, an issue that has a significant effect on the adsorption efficiency towards AO dye.

On the other hand, Fig. 6b shows the spectrum of TTWM500 after adsorption of AO. As shown, the spectrum indicates some changes compared to the spectra of free AO, and TTWM500 before adsorption. These changes include alterations in intensities, shifts in position, and even complete disappearance of the band. For example, spectrum of free AO (Fig. 6b) shows a band at 2928 cm^−1^ which could be ascribed to the C-H stretching vibration and a broad peak in the range of 2750–3450 cm^−1^ which could be assigned to -NH^+^ of AO. These two bands almost disappear in the spectrum after adsorption. Similarly, the spectrum of free AO shows a band in the range of 1573–1590 cm^−1^ which could be attributed to the skeletal vibration of the phenyl ring of AO. These bands shift to 1562 cm^−1^ in the spectrum of TTWM500 after adsorption (this peak was not in the spectrum of TTWM500 before adsorption). The sharp peak at 1497 cm^−1^ which can be assigned to the aliphatic CN stretching coupled to the aromatic C=C (in ring) vibrations is absent in the spectrum after adsorption. Instead, a small peak at 1501 cm^−1^ appears, which could be a confirmation for the existence of AOH^+^ on the surface of the adsorbent. A similar observation could be reported for the peak at 1633 cm^−1^ in the spectrum of AO which could be ascribed to the C-C stretching band and the scissoring band of N-H. Upon adsorption, the later peak disappears. The sharp peak at 1166 cm^−1^ in the spectrum of free AO which could be due to the in-plane C-H and N-H bending shifts to 1160 cm^−1^ with much less intensity and sharpness. The peak at 919 cm^−1^ which might be due to the rocking of N-H shifts to 883 cm^−1^ following adsorption. The medium peak at 696 cm^−1^ which could be due to N-H twisting disappears upon adsorption.

These findings and the observed shift in the N-H vibrational frequencies following the adsorption and compared to the free AO suggest the occurrence of an interaction between the positively charged NH^+^ moiety of AO (central phenyl ring) and the surface of the TTWM500 (El-Azazy et al. 2021; Karmakar et al. 2019; Sharma and Ilanchelian 2014).

**Fig. 7:**
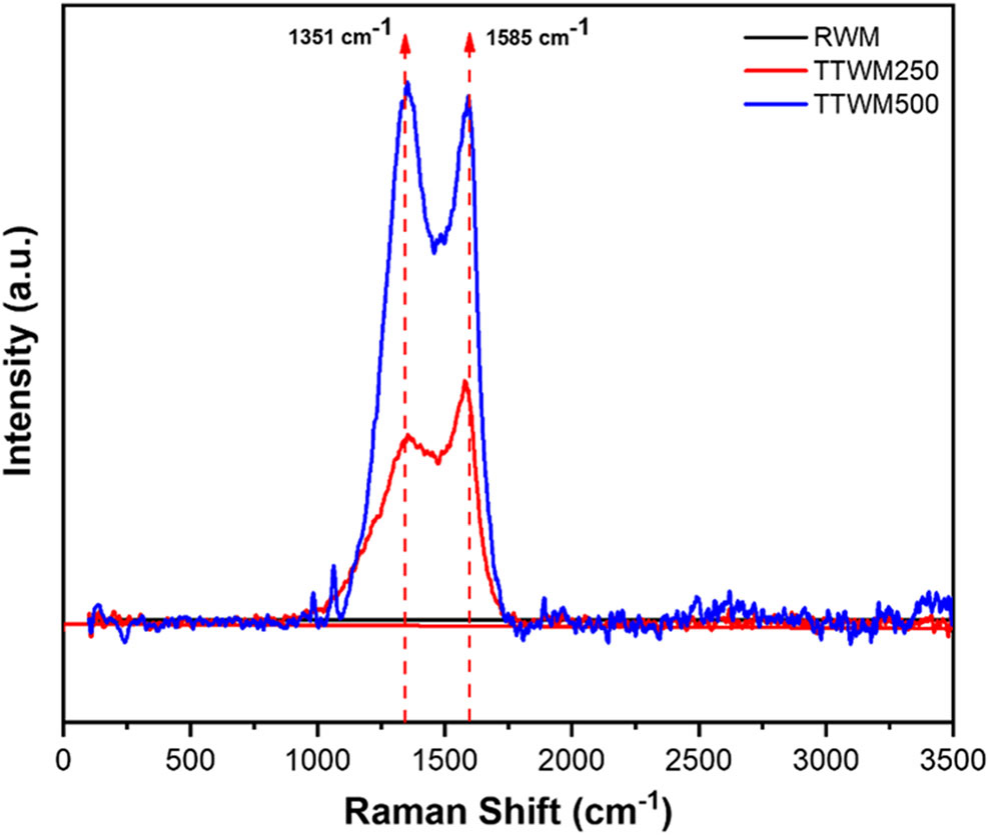
Raman spectra of the three adsorbents

Figure 7 shows the Raman spectra of raw as well as the biochars. Spectra of TTWM at both temperatures, and compared to the RWM, show significant D- and G-bands at 1351 and 1585 cm^−1^, respectively. Both bands are unique peaks in the spectra of carbonaceous materials. While the D-band reveals the carbon lattice properties such as defects and sizes (not the composition of the carbonaceous material), the G-band represents the stretching of C-C in the *sp*^2^ system (Stankovich et al. 2007; Childres et al. 2013). Moreover, the intensity ratio *I*_*D*_:*I*_*G*_ has increased from TTWM250 (0.77) to TTMW500 (1.053), which might have occurred due to the impact of thermal treatment on increasing the number of defects in the *sp*^2^ plane of the carbon of biochars. Collected data prove the formation of carbonaceous materials with higher defects after thermal treatment compared to the raw sample.

#### Thermo-gravimetric analysis (TGA)

**Fig. 8:**
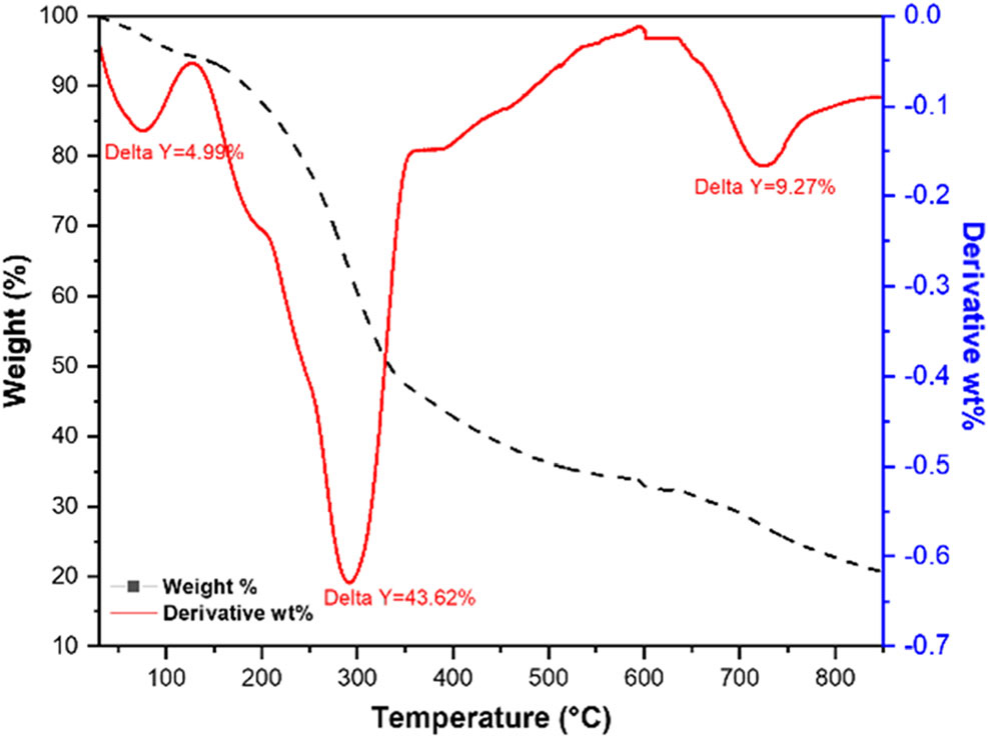
TGA/*d*TGA analysis of RWM

The data shown in Fig. 8 is the TGA analysis of RWM obtained under N_2_ and with a heating rate of 10 °C/min. The obtained data show that the weight loss in WMR samples has occurred over two main steps: (1) loss of physically adsorbed water in the range of at 25−130 °C and (2) between 150 and 800 °C where more than 50% of the sample is decomposed at this stage. This step is indicated by one large peak at 295 °C, a small peak at 727 °C, and a shoulder at 193.5 °C and could have occurred due to the decomposition of organic matter and the formation of thermally stable carbonaceous material (Chaudhari and Singhal 2015).

### Equilibrium isotherms

Adsorption isotherm models were used to study the behavior of AO adsorption onto TTWM500. In this study, four models were used: Langmuir, Freundlich, Temkin, and Dubinin-Radushkevich (D-R) (Benkaddour et al. 2018**;** Langmuir 1918**;** Guo and Wang 2019**;** Araújo et al. 2018**;** Moussavi and Barikbin 2010).

**Fig. 9:**
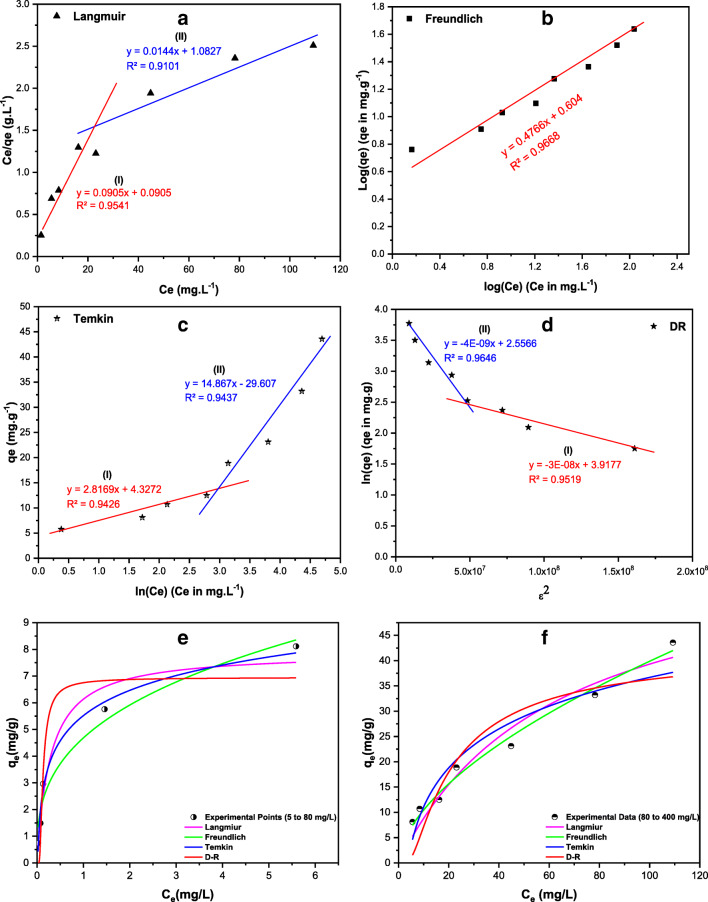
Adsorption linear models of AO onto TTWM500 including Langmuir (**a**), Freundlich (**b**), Temkin (**c**), DR (**d**), and nonlinear models at low (**e**) and high AO concentrations (**f**)

Figure 9a–f and Table 6 show the obtained data for each of the proposed models employing both linear and nonlinear fitting. As shown in Table 6, and implementing the linear fitting, Freundlich model had the highest goodness-of-fit among the four isotherm models (*R*^2^, coefficient of determination value: 0.967 (Freundlich) > 0.965–0.952 (D-R) > ~ 0.944 (Temkin) > 0.910 (Langmuir)), suggesting the occurrence of multilayer distribution of the AO onto the heterogenous surface of TTWM500 with an interaction among the AO molecules.

**Table 6:** General equations of Langmuir, Freundlich, Temkin, and Dubinin-Radushkevich (D-R) isotherms for the adsorption of AO onto TTWM500 and their fitting parameters

Model	Type and model equation	Parameter	DC (mg/L)	
			5–80	80–400
Langmuir	Linear $\frac{C_e}{q_e}=\frac{1}{q_m\ {K}_L}+\frac{C_e}{q_m}$	*q*_max_ (mg.g^−1^)	11.05	69.44
		*K*_*L*_ (L.mole^−1^)	0.99	0.01
		*R*^2^	0.954	0.910
	Nonlinear ${q}_e=\frac{q_m\ {K}_L\ {C}_e}{1+{K}_L\ {C}_e}$	*q*_max_ (mg.g^−1^)	7.90	63.95
		*K*_*L*_ (L.mole^−1^)	3.47	0.016
		*R*^2^	0.965	0.953
Freundlich	Linear log(*q*_*e*_) $=\log \left({K}_F\right)+\left(\frac{1}{n}\right)\log \left({C}_e\right)$	*K*_*F*_ (mole/g) (L/mole)^1/*n*^	4.02	
		1/*n*	0.477	
		*R*^2^	0.967	
	Nonlinear ${q}_e={K}_F{C}_e^{\frac{1}{n}}$	*K*_*F*_ (mole/g) (L/mole)^1/*n*^	4.68	2.74
		1/*n*	0.34	0.58
		*R*^2^	0.972	0.985
Temkin	Linear ${q}_e=\frac{RT}{b_T}\ln \left({A}_T\right)+\frac{RT}{b_T}\ln \left({C}_e\right)$	b_*T*_ (J/mole)	864.8	163.8
		A_*T*_ (L/mole)	4.65	0.136
		*R*^2^	0.943	0.944
	Nonlinear ${q}_e=\frac{RT}{b_T}\ \ln \left({A}_T\ {C}_e\right)$	b_*T*_ (J/mole)	1813.8	222.81
		A_*T*_ (L/mole)	56.79	0.271
		*R*^2^	0.958	0.911
D–R	Linear ln(*q*_*e*_) = ln(*q*_*s*_) − *βϵ*^2^ $$ \epsilon = RT\left(1+\frac{1}{C_e}\right) $$ $$ E=\raisebox{1ex}{$1$}\!\left/ \!\raisebox{-1ex}{$\sqrt{2\beta }$}\right. $$	*Β*	4×10^−9^	3×10^−8^
		E (kJ/mole)	11.18	4.08
		q_*s*_ (mg/g)	12.89	50.28
		*R*^2^	0.965	0.952
	Nonlinear *q*_*e*_ = *q*_*s*_ . exp (−*β*. *ϵ*^2^)	*Β*	6.64×10^−9^	1.39×10^−6^
		E (kJ/mole)	8.67	1.90
		q_*s*_ (mg/g)	7.03	43.07
		*R*^2^	0.899	0.835

*q*_*e*_, amount of adsorbate in the adsorbent at equilibrium; *K*_*L*_, Langmuir isotherm constant; *q*_*max*_, maximum monolayer coverage capacities; *K*_*F*_, Freundlich adsorption constant; ***C***_*e*_ equilibrium concentration; *q*_*s*_, theoretical isotherm saturation capacity; *A*_*T*_, Temkin isotherm equilibrium binding constant; *R*, universal gas constant (8.314 J/mol K); *T* is the temperature (K); *b*_*T*_, Temkin isotherm constant

For Freundlich isotherm, the constants *K*_*F*_ and 1/*n* are indicators for the adsorbent capacity and change in the intensity of the adsorption as well as the deviation from linearity (Al-Saad et al. 2019). In general, when the value of *n* is > 1, the adsorption is favorable. Table 6 shows that the values of *n* and 1/*n*, following linear fitting, are 2.098 and 0.477, respectively, indicating that approximately 47% of adsorption took place on the active sites. By applying the nonlinear fitting, the values of *n* and 1/*n* were 1.724 and 0.58 at higher concentrations (80 to 400 mg/L), while for the lower AO concentrations (5 to 80 mg/L), *n* and 1/*n* were 2.941 and 0.34, respectively. Therefore, it could be concluded that adsorption of AO onto TTWM500 was favorable at all concentrations.

The Langmuir model (Fig. 9a, e, f) suggests the occurrence of monolayer adsorption on the homogenous surface of the adsorbent. Moreover, it assumes the existence of finite number of adsorption sites and that no interaction is taking place between the adsorbate molecules. Langmuir model is used to obtain the maximum adsorption capacity (*q*_max_). Table 6 shows that the value of *q*_max_ obtained from linear Langmuir (two linear segments) is 69.44 mg/g (compared to 63.95 mg/g using nonlinear fitting), suggesting monolayer coverage over homogenous adsorption sites within TTWM500. On the other hand, the *R*_*L*_ value (separation factor) can be calculated using Eq. 5:

5 $$ {R}_L=\frac{1}{1+{K}_L\left({C}_0\right)} $$

In the above dimensionless equation, *K*_*L*_ denotes the Langmuir isotherm constant (L.mole^−1^), while *C*_0_ is the initial concentration (mg.L^**−**1^). The adsorption is favorable when *R*_*L*_’s value is between 0 and 1 (0 < *R*_*L*_ < 1). However, if the *R*_*L*_ value is >1, then the adsorption process is unfavorable. Also, the adsorption process is considered irreversible if the value of *R*_*L*_ = 0. Consequently, the *R*_*L*_ value measured for TTWM500 was < 1 and > 0, demonstrating favorable adsorption of AO onto TTWM500 (Langmuir 1918; Guo and Wang 2019). Yet, Langmuir isotherm could not be used to explain the overall adsorption of AO onto TTWM500 as indicated by the *R*^2^ value.

Linear and nonlinear Temkin isotherms are shown in Fig. 9c, e, f. Temkin model is used to reflect the adsorbate–adsorbent interaction, where the heat of adsorption of the molecules in a layer decreases linearly with the adsorbate–adsorbent interactions. Table 6 shows that the sorption energy in linear Temkin isotherm at low AO concentrations was 864.8 J/mol compared to 163.8 J/mol at high concentration. For the nonlinear isotherm, the sorption energy at low and high concentrations of AO was 1813 and 222.8 J/mol, respectively.

Finally, the D-R isotherm (Fig. 9d–f) was utilized to identify the adsorption mechanism based on the free energy value. Physical adsorption occurs when free energy is ˂ 8.0 kJ/mol, while chemical adsorption occurs when free energy is ˃ 8.0 kJ/mol. The free energy for adsorption of AO onto TTWM500, as revealed in Table 6, shows two types of adsorption mechanisms: the chemisorption mechanism (free energy = 11.18 kJ/mol for linear and 8.67 kJ/mol for nonlinear model) at low concentrations of AO and physisorption (free energy = 4.08 and 1.90 kJ/mol for linear and nonlinear models, respectively) at high concentrations of AO. This finding shows that the adsorption of AO onto TTWM500 runs over two stages; the first stage could be attributed to chemical adsorption to form one layer. The second could be attributed to the physical interactions to form multilayers. The *q*_max_ for the low concentration region was 12.89 and 7.03 mg/g for the linear and nonlinear models, respectively. At high concentration, the *q*_max_ was 50.28 and 43.07 mg/g for both models.

All in all, adsorption of AO onto TTWM500 agreed well with Freundlich isotherm. However, prevalence of multilayer adsorption might not be the exclusive mechanism. Taking into consideration the findings of adsorbents’ characterization, output of BB design, and the results of the equilibrium study, adsorption of AO onto TTWM500 can be better described. As per the findings of the D-R isotherm, adsorption of AO onto TTWM500 follows two patterns depending on the DC. Occurrence of chemisorption at low AO concentrations could be explained considering both the nature of AO under the experimental conditions and the adsorbent structure as revealed by FT-IR, SEM, and BET analyses. At low concentrations of the dye, the adsorption of a monolayer of AO onto the surface of TTWM500 and the fixation of the dye inside the adsorbent pores via hydrogen bonding (through the nitrogen moieties in AO) and electrostatic interactions is probably the mechanism. On the other hand, AO possesses a conjugated system (Scheme 1). As per Raman spectroscopic analysis, the biochar, TTWM500 has a graphene like structure with *sp*^2^ system. These findings corroborate the occurrence of π-π electron donor-acceptor (EDA) interactions between AO and the carbonaceous adsorbent.

As per the BET analysis, the surface area of TTWM500 is low, an issue that could not support the achieved %*R* and *q*_*e*_ values. However, the porous structure of the carbonaceous adsorbent could be the justification. The presence of mesopores and macropores with an advanced intra- and multilayered pore structure on the surface of TTWM500 could explain the subsequent physical interactions to form multilayers. This finding comes in alignment with the equilibrium isotherm finding where Freundlich isotherm was a perfect fit to explain the adsorption behavior of AO onto TTWM500.

### Kinetic studies

**Fig. 10:**
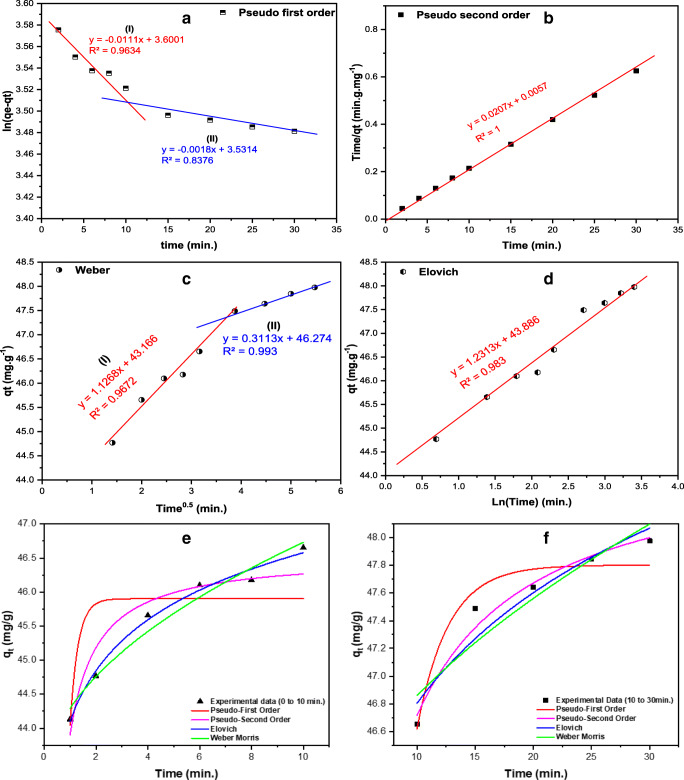
Kinetic models for the adsorption of AO onto TTWM500. The panels a-d show the linear fitting of data: **a** Pseudo-first order (PFO), **b** Pseudo-second order, **c** Weber–Morris (WM), and **d** Elovich. The panels **e**, and **f** show the nonlinear fitting of data between: **e** 0-10 min and **f** 10-30 min

The kinetics of the adsorption of AO dye onto TTWM500 was studied using linear and nonlinear fitting of the following models: pseudo-first order (PFO), pseudo-second order (PSO), Elovich, and Weber–Morris (WM) (Benkaddour et al. 2018; Hameed et al. 2009). The linear kinetic models in Figs. 10a and 10b show a representation of ln (*q*_*e*_−*q*_*t*_) and time/*q*_*t*_ versus time and for the PFO and PSO kinetic models. According to the data presented in Table 7 and by comparing the *R*^2^ values for the four models, adsorption of AO onto the studied adsorbent could be best described using the PSO model, where the *R*^2^ = 1.000. The nonlinear fitting (Fig. 10e, f) shows similar data to the linear models with the highest *R*^2^ value (0.976) being obtained for PSO at reaction time between 10 and 30 min. Therefore, the adsorption reaction according to the linear models could be represented as follows:

6 $$ \mathrm{AO}+\mathrm{TTWM}500\left(\overset{k}{\to}\right)\kern0.5em \left\{\mathrm{AO}-\mathrm{TTWM}500\right\} $$

**Table 7:** The kinetics study results corresponding to the data shown in Fig. 10

Model	**Type and** model equation	Parameter	Time (min)	
			0–10	10–30
PFO	Linear ln(*q*_*e*_ − *q*_*t*_) = ln(*q*_*e*_) − *k*_1_*t*	*K*_1_ (min^−1^)	0.011	0.002
		*q*_*e*_ (mg/g)	36.60	34.17
		*R*^2^	0.963	0.838
	Nonlinear $\frac{d{q}_t}{dt}$*= k*_1_(*q*_*e*_−*q*_*t*_)	*K*_1_ (min^−1^)	3.207	0.369
		*q*_*e*_ (mg/g)	45.90	47.80
		*R*^2^	0.589	0.937
PSO	Linear $\frac{t}{qe}=\frac{1}{k_2{q}_e^2}+\frac{1}{q_e}$*t*	*K*_2_ (g.mg^−1^.min^−1^)	0.075	
		*q*_*e*_ (mg/g)	48.31	
		*R*^2^	1.000	
	Nonlinear $\frac{d{q}_t}{dt}$*= k*_2_(*q*_*e*_−*q*_*t*_)^2^	*K*_2_ (g.mg^−1^.min^−1^)	0.3565	0.049
		*q*_*e*_ (mg/g)	46.54	48.67
		*R*^2^	0.908	0.976
Elovich	Linear ${q}_t=\frac{1}{\beta}\ln \left(\alpha \beta \right)+\frac{1}{\beta}\ln (t)$	*α*	3.7 × 10^15^	
		*β*	0.812	
		*R*^2^	0.983	
	Nonlinear *q*_*t*_*=* $\frac{1}{\beta}\times \ln \left(1+\alpha \beta t\right)$	*α*	6.9 × 10^17^	4.9 × 10^16^
		*β*	0.930	0.867
		*R*^2^	0.909	0.925
WM	Linear *q*_*t*_ = *K*_*I*_*t*^0.5^ + *C*	*K*_*I*_	1.127	0.311
		*C*	43.17	46.27
		*R*^2^	0.967	0.993
	Nonlinear *q*_*t*_ = *K*_*I*_*t*^0.5^ + *C*	*K*_*I*_	1.127	0.535
		*C*	43.17	45.17
		*R*^2^	0.967	0.883

where *q*_*t*_ is adsorbed quantity at time *t*; while *α* and *β* are initial sorption concentration rate (mg.g^−1^.min^−1^) and desorption constant (g/mg), *K*_*I*_ is intraparticle diffusion rate constant (mg.g^−1^.min^−0.5^), and *C* is boundary thickness effect

Accordingly, the rate of the reaction = *k*[AO][TTWM500], indicating that the adsorption rate depends on both the adsorbate and the adsorbent. In addition, the adsorbed quantity determined from the nonlinear model was between 46.54 and 48.67 mg/g, similar to the obtained data from the equilibrium isotherms.

The Weber–Morris (WM) intraparticle diffusion model (Fig. 10c, e, f) reveals significant findings, where, besides the intra-particle diffusion, there is another mechanism that controls the diffusion of AO. According to the calculated parameters in Table 7 from linear and nonlinear models, diffusion occurs over two stages. The adsorption reaction commenced with a high intraparticle diffusion rate (1.127 mg.g^−1^.min^−0.5^) with high boundary layer thickness (43.17 mg/g). The intraparticle diffusion rate decreased with time (0.3113 and 0.535 mg. g^−1^.min^−0.5^ for linear and nonlinear fittings, respectively) when the boundary layer became 46.27 and 45.17 mg/g for linear and nonlinear models, respectively. On the other hand, in the Elovich model (Fig. 10d–f), the initial adsorption of AO onto TTWM500 was very high in the case of both fittings and equals 3.7 × 10^15^ mg.g^−1^.min^−1^ for linear models, and the nonlinear model ranged between 6.9 × 10^17^ and 4.9 × 10^16^ mg.g^−1^.min^−1^, justifying the elevated adsorption efficiency of TTWM500.

### Desorption and regeneration studies

**Fig. 11:**
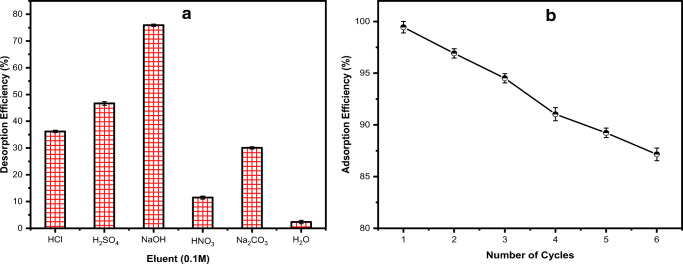
**a** Effect of eluent type on the AO desorption and **b** the regeneration performance of the TTWM500 adsorbent towards AO (using 0.1 M NaOH as an eluent for 6 cycles)

Adsorbent regeneration and reusability represent the most important aspects of the adsorption study. In other words, and to make the adsorption process efficient and cost-effective, it is crucial to reuse the adsorbent following multiple sorption/desorption cycles (Singh and Singhal 2015). For this purpose, a desorption study with six different eluents was conducted, followed by six consecutive sorption-desorption cycles. The obtained data (Fig. 11a) show a comparison between the utilized eluents and their corresponding desorption efficiency (%). As per the shown data, 0.1 M NaOH had the highest desorption efficiency of 75.90%, followed by 0.1 M H_2_SO_4_, which showed a desorption efficiency of 46.65%. On the other hand, H_2_O and 0.1 M HNO_3_ showed the lowest desorption efficiency (2.31% and 11.46%, respectively). Therefore, 0.1 M NaOH was used as the most suitable desorbing agent.

For the adsorbent regeneration, the cyclic sorption-desorption findings shown in Fig. 11b illustrate that the dye removal efficiency has decreased slightly from 99.45% (cycle 1) to 87.15% (cycle 6), probably due to the incomplete desorption and removal of AO from the active adsorption sites. The average AO removal efficiency was around 87% over the six consecutive regeneration cycles. The obtained results reveal an excellent reusability of TTWM500 and the possibility of its regeneration even after a larger number of cycles.

## Conclusions

Watermelon rinds (WMR) have been developed and effectively utilized as a green, low-cost, non-conventional adsorbent to remove AO dye from contaminated water samples. Both raw and thermally treated rinds at 250 and 500 °C were tested for AO removal, and TTWM500 showed the best adsorption efficiency. The effect of four different variables, namely pH, AD, DC, and ST on the removal efficiency of TTWM500 was tested. The Box–Behnken design was applied for analyzing and modelling the adsorption process. Results showed that the obtained regression models could properly rationalize the experimental findings. The three adsorbents were characterized using FT-IR, Raman, BET, SEM, and TGA analyses. The obtained data showed that the surface area of TTWM500 was the highest among the tested adsorbents (5.03 m^2^/g), and the SEM micrographs showed the presence of large pores in the TTWM500 sample. Furthermore, the FT-IR analysis before and after adsorption showed the alterations in intensities, shifts in position, and even complete disappearance of some functional groups confirming the interaction of AO and the adsorbent. Equilibrium studies using linear and nonlinear fittings showed that data fit well to Freundlich isotherm and that adsorption is favorable with a maximum adsorption capacity of 69.44 mg/g from the linear fitting Langmuir equation. Besides, the adsorption is chemisorption and physisorption at low and high AO concentrations, respectively. The kinetic studies showed that the adsorption occurred over two stages with a high intraparticle diffusion rate (1.127 mg. g^−1^. min^−0.5^) and high boundary layer thickness (43.17 mg/g). The desorption study showed that TTWM500 could be regenerated with the adsorption efficiency being preserved up to 87% after six cycles.

## Supplementary Information

ESM 1 (DOCX 17 kb)

## Data Availability

All data used to support the findings of this study are included within the article.
