# Supplementary material for: Watermelon rinds as cost-efficient adsorbent for acridine orange: a response surface methodological approach
Source: Environ Sci Pollut Res Int. 2021 Apr 8;30(28):71554–73. doi: 10.1007/s11356-021-13652-9 (PMC10257614; doi:10.1007/s11356-021-13652-9)
Supplement: Supplementary file 1 — (DOCX 17 kb) [file 11356_2021_13652_MOESM1_ESM.docx]

Watermelon Rinds as Cost-efficient Adsorbent for Acridine Orange: A Response Surface Methodological Approach

Ahmed S. El-Shafie ^a^, Siham S. Hassan ^a^, Nuri Akther ^a^ and Marwa El-Azazy ^a,^*

^a^ Department of Chemistry and Earth Sciences, College of Arts and Sciences, Qatar University, Doha 2713, Qatar.

* Corresponding author at: Department of Chemistry and Earth Sciences, Qatar University, Doha 2713, Qatar. Tel.: +974 4403 4675; Fax: +974 4403 4651.

E-mail address: marwasaid@qu.edu.qa (Dr. Marwa Said)

**Table S1** Comparison between the performances of WMR (current investigation) and different WM-based adsorbents as reported in literature.

| **Adsorbent** | **Modification Method** | **Analytical Approach Used** | **BET Surface Area (m^2^/g)** | **Adsorbate** | ***q_max_* (mg/g)** | **%R** | **Ref.** |
| --- | --- | --- | --- | --- | --- | --- | --- |
| TTWM500 | Please see the experimental section of the current approach | Multivariate analysis – BB design | 5.03 | Acridine orange (AO) | 69.44 | 99.4% | Current work |
| Titanium oxide nanoparticles (NPs) loaded onto activated carbon (AC) prepared from bio-waste watermelon rind (TiO_2_–NPs–ACWR) | AC was prepared from bio-waste WR. The TiO_2_–NPs were synthesized through a hydrothermal method (AC was mixed with TiO_2_ at a ratio of 1:10, followed by stirring at room temperature for 20 h). The produced TiO_2_–NPs were loaded on ACWR, washed, and dried at the temperature of 70 ˚C for 3 h. | Response surface methodology based on central composite design (CCD) | 667.8 | Congo red (CR) Phenol red (Ph R) | Ph R: 55.6 DR: 17 | Ph R: 100.24%  DR: 101.45% | (Masoudian et al 2019) |
| Saponified watermelon shell (SWS)  Acidified watermelon shell (AWS) | **SWS:** Watermelon shells were washed, cut into small pieces, and mixed with 5 g of Ca(OH)_2_. The mixture was then agitated for 12 h, followed by filtration and drying at 100 ˚C overnight.  **AWS:** Watermelon shells were dried in the air, heated in an oven at 120 ˚C, and then blended with 0.1 M citric acid. | OFAT* | ND** | Cu (II) | SWS: 31.25  AWS: 27.03 | ND** | (Gupta and Gogate 2016) |
| Untreated watermelon peels (UTWMP)  Thermally treated watermelon peels (TWMP) | Watermelon peels washed, dried at 110 ˚C, and sieved to produce UTWMP. The obtained material was then treated by 0.1M HNO_3_ and then thermally treated in a closed furnace to have TWMP. | OFAT* | UTWMP: 15.1  TTWMP: 23.4 | Methyl parathion pesticide (MP) | 24.3 µmol/g | 99.0% | (Memon et al 2008) |
| Biochar derived from watermelon rinds (PB-400, PB-500, PB-600, MB-HCl, and MB-KOH) | Watermelon rinds were washed and dried at85◦C for 24 h. The resulted biochar was burnt at 400 ˚C, 500 ˚C, and 600 ˚C (PB–400, PB–500, and PB–600). PB–500 was then modified into MB-HCl and MB-KOH. | OFAT* | PB-400: 3.13  PB-500: 4.75  PB-600: 3.87  MB-HCl: 14.2  MB-KOH: 13.8 | Tl (I) | PB-500: 178.4 | 98.5% | (Li et al 2019) |
| Treated watermelon seeds (WST) | Watermelon seeds were washed and dried at room temperature for 24 h. Dried samples were treated with hexane and then crushed and placed in an oven at 80 ˚C for 8 h. | OFAT* | 2 | Reactive yellow 145 (RY145) | 115 | 84% | (Benkaddour et al 2018) |

*OFAT: One -Factor-at-a-time, ** ND: Not determined.
